# Baxdrostat: A First-in-Class Aldosterone Synthase Inhibitor for Resistant Hypertension

**DOI:** 10.1021/acsptsci.6c00102

**Published:** 2026-03-20

**Authors:** Imma Capriello, Alexander Dömling

**Affiliations:** † Czech Advanced Technology and Research Institute (CATRIN), Palacký University Olomouc, Slechtitelů 27, 77900 Olomouc, Czech Republic; ‡ Institute of Molecular and Translational Medicine, Faculty of Medicine and Dentistry, Palacký University and University Hospital Olomouc, Hněvotínská 1333/5, Olomouc 77900, Czech Republic

**Keywords:** aldosterone synthase, Baxdrostat, resistant hypertension, CYP11B2, structure-based drug design, cardiovascular pharmacology

## Abstract

Hypertension remains the world’s leading preventable cause of cardiovascular morbidity and mortality. Despite the availability of diverse antihypertensive drug classes, resistant hypertension continues to affect millions globally, leading to a disproportionate risk of stroke, heart failure, kidney disease, and premature death. Aldosterone excess is a central driver of treatment resistance, yet direct pharmacological suppression of aldosterone biosynthesis has long eluded clinical success due to the challenge of selectively targeting aldosterone synthase (CYP11B2) over its near-identical paralog 11β-hydroxylase (CYP11B1). Baxdrostat (CIN-107, RO6836191), an orally bioavailable, highly selective aldosterone synthase inhibitor (ASI), has now demonstrated robust blood pressure reduction in phase 3 clinical trials, marking a potential paradigm shift in the management of resistant hypertension. This Review summarizes the pathophysiology of aldosterone in hypertension, the molecular pharmacology of CYP11B2 inhibition, the discovery and development of Baxdrostat, and its clinical evaluation. We further discuss the broader implications of targeting steroidogenic cytochrome P450 enzymes and highlight future opportunities and challenges as Baxdrostat and related agents enter the cardiovascular pharmacopeia.

## Graphical abstract

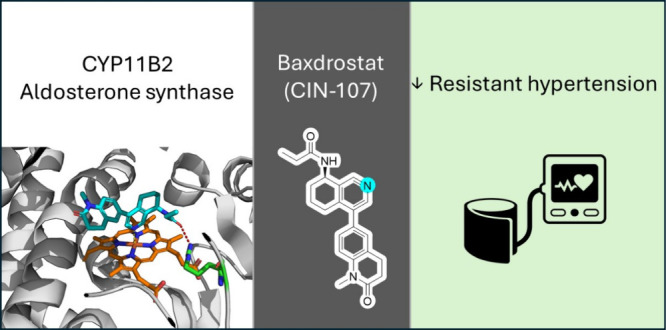

Hypertension is a cardiovascular disorder defined by a sustained elevation of arterial blood pressure above physiological thresholds. It is the most prevalent circulatory disease worldwide, affecting more than one billion people (approximately 18% of the global population),^1^ with incidence rising due to population aging, sedentary lifestyles, obesity, and excessive dietary sodium intake. Despite the availability of multiple effective pharmacological classes, optimal blood pressure control is achieved in fewer than 20% of patients.^2–4^ While most cases are classified as essential hypertension, secondary forms such as renal parenchymal disease, renovascular hypertension, and primary aldosteronism represent an underrecognized subset. Particularly challenging is the primary aldosteronism, resulting from autonomous aldosterone overproduction by the adrenal cortex, and accounting for 15–20% of cases of resistant hypertension.^5,6^ The consequences of persistent elevation in blood pressure are profound. Hypertension, in fact, remains the leading global risk factor for stroke, myocardial infarction, heart failure, and chronic kidney disease, contributing to more than ten million deaths each year.^7,8^ Beyond the human toll, the economic impact is substantial, encompassing both direct healthcare costs and loss of productivity.^9^ Growing evidence underscores the need for targeted therapeutic strategies aimed at modulating aldosterone synthesis or action to improve blood pressure control and prevent end-organ damage.

## Current Therapies and Their Limitations

The pharmacological landscape of antihypertensive therapy is broad encompassing centrally acting agents (e.g., clonidine **1**), renin–angiotensin system inhibitors (e.g., captopril **2** and valsartan **3**), calcium channel blockers (e.g., nifedipine **4**), β-adrenergic antagonists (e.g., propranolol **5**), renin inhibitors (e.g., aliskiren **6**) and diuretics (e.g., furosemide **7**) (Figure 1). Clinical guidelines generally recommend combinations of these drug classes, most frequently angiotensin-converting enzyme inhibitors or angiotensin receptor blockers, together with a calcium channel blocker and a thiazide or thiazide-like diuretic. While this regimen achieves adequate blood pressure control in a large proportion of patients, a significant minority remains uncontrolled despite adherence to triple therapy. For patients with resistant hypertension, the addition of a mineralocorticoid receptor antagonist, such as spironolactone or eplerenone, has long been the recommended next step. These agents can be highly effective in lowering blood pressure by antagonizing the effects of aldosterone on its receptor. However, their clinical utility is often limited by poor tolerability and adverse events. Spironolactone, for example, may induce gynecomastia or menstrual irregularities due to its nonselective steroidal activity, while both spironolactone and eplerenone are associated with a clinically relevant risk of hyperkalemia, especially in patients with renal impairment.^10–13^ Moreover, mineralocorticoid receptor antagonists do not prevent aldosterone biosynthesis itself, and paradoxically lead to compensatory increases in circulating aldosterone and renin concentrations. This feedback activation has raised concerns that receptor-independent actions of aldosterone, including pro-fibrotic and pro-inflammatory effects, may remain unmitigated. Collectively, these limitations highlight the need for therapies that intervene earlier in the aldosterone pathway, suppressing hormone synthesis rather than merely antagonizing receptor binding.

**Figure 1:**
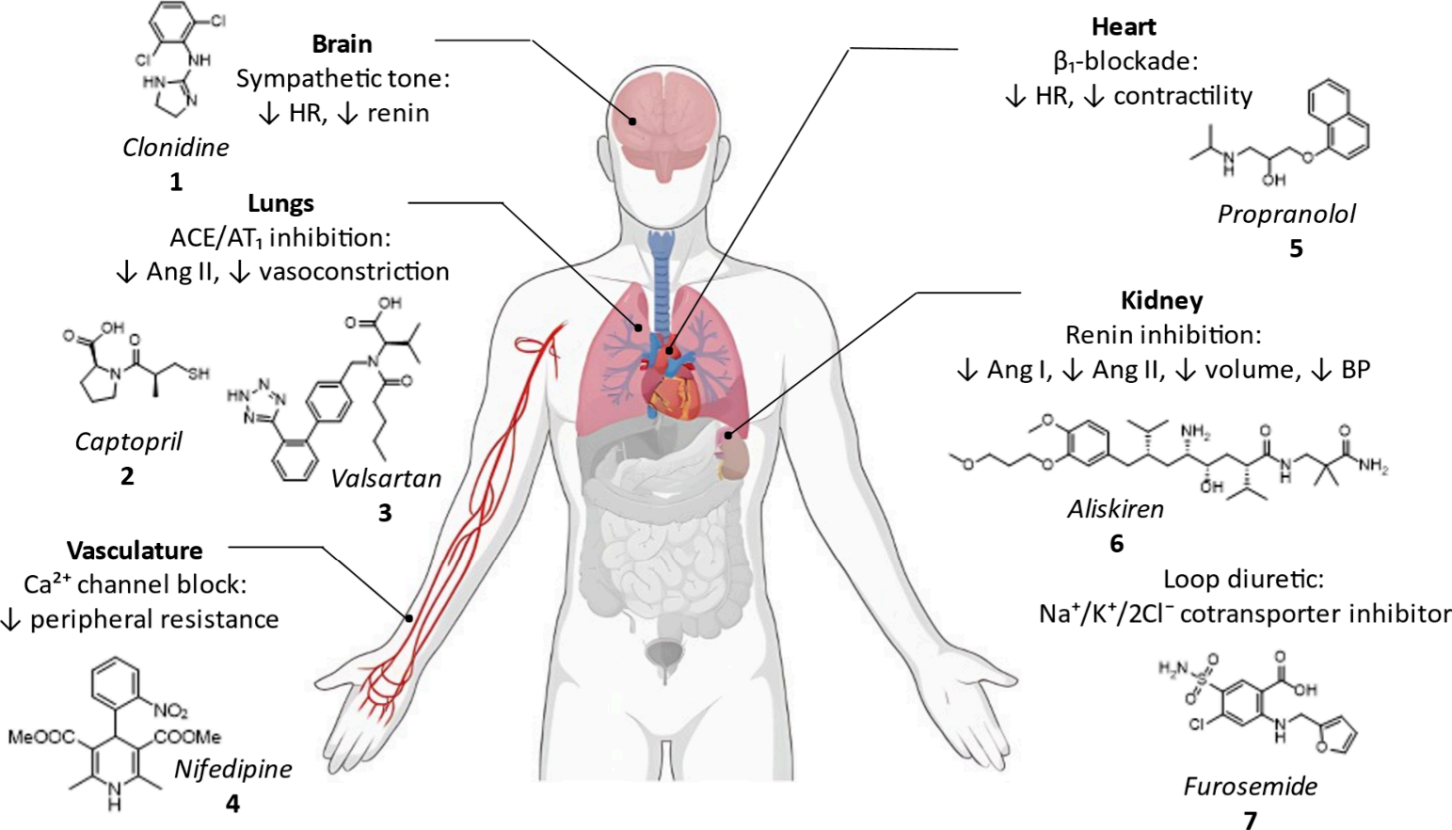
Sites and mechanisms of action of major antihypertensive drug classes to lower blood pressure.

## Molecular Mechanisms: Aldosterone and Blood Pressure Regulation

Aldosterone acts as the final mediator of the renin–angiotensin–aldosterone system (RAAS), a finely tuned endocrine mechanism responsible for regulating blood volume and electrolyte balance. Revisiting this cascade provides insight into the limitations of current therapies and the rationale for selectively targeting aldosterone synthesis rather than merely blocking its receptor.^14^ The RAAS cascade is initiated by the juxtaglomerular cells of the kidney, which, upon sensing a decline in blood pressure, secrete renin into the circulation. Renin catalyzes the conversion of the plasma protein angiotensinogen into angiotensin I, which is subsequently cleaved to angiotensin II by angiotensin-converting enzyme (ACE). Angiotensin II is a potent vasoconstrictor and a key stimulator of aldosterone release: by acting on zona glomerulosa cells in the adrenal cortex, it upregulates CYP11B2 expression and activity, thereby promoting the final hydroxylation and oxidation steps that convert 11-deoxycorticosterone into aldosterone (Figure 2). Aldosterone binds to intracellular mineralocorticoid receptors in the principal cells of the distal nephron, inducing the transcription of transport proteins such as the epithelial sodium channel (ENaC) on the apical membrane and the Na^+^/K^+^-ATPase on the basolateral membrane. While ENaC facilitates sodium entry from the tubular lumen, Na^+^/K^+^-ATPase drives sodium reabsorption into the bloodstream in exchange for potassium uptake. This coordinated activity enhances sodium and water retention—by osmotic coupling—thereby increasing blood volume and pressure while simultaneously promoting potassium excretion. Beyond its renal actions, aldosterone contributes to maladaptive cardiovascular remodeling. Elevated aldosterone levels are associated with increased vascular stiffness, endothelial dysfunction, oxidative stress, inflammation, and fibrosis within cardiac and renal tissues.^15^ Consequently, excess of aldosterone is now recognized not only as a mediator of resistant hypertension but also as a driver of heart failure, chronic kidney disease, and primary aldosteronism. Notably, up to one in five patients with resistant hypertension exhibit biochemical or clinical evidence of primary aldosteronism, underscoring the role of aldosterone dysregulation in difficult-to-control blood pressure.^16^ Aldosterone is synthesized from cholesterol through a cascade of steroidogenic enzymes, with CYP11B2 (aldosterone synthase) catalyzing the final steps (Figure 2). As a member of the cytochrome P450 superfamily, which comprises more than 50 human isoforms with highly conserved catalytic domains, CYP11B2 poses a formidable challenge for drug discovery. The main difficulty lies in achieving sufficient selectivity to prevent off-target inhibition. Notably, CYP11B2 shares 97% amino acid homology in its active site with its paralog CYP11B1, the enzyme responsible for cortisol synthesis. Inhibiting CYP11B1 inadvertently suppresses cortisol production, potentially leading to adrenal insufficiency and systemic toxicity. Moreover, interference with other P450 enzymes involved in steroidogenesis or xenobiotic metabolism can trigger electrolyte disturbances, metabolic derangements, or hepatotoxicity. Therefore, the pursuit of selective CYP11B2 inhibition requires exceptional molecular precision to avoid collateral disruption of related biochemical pathways.^17–19^

**Figure 2:**
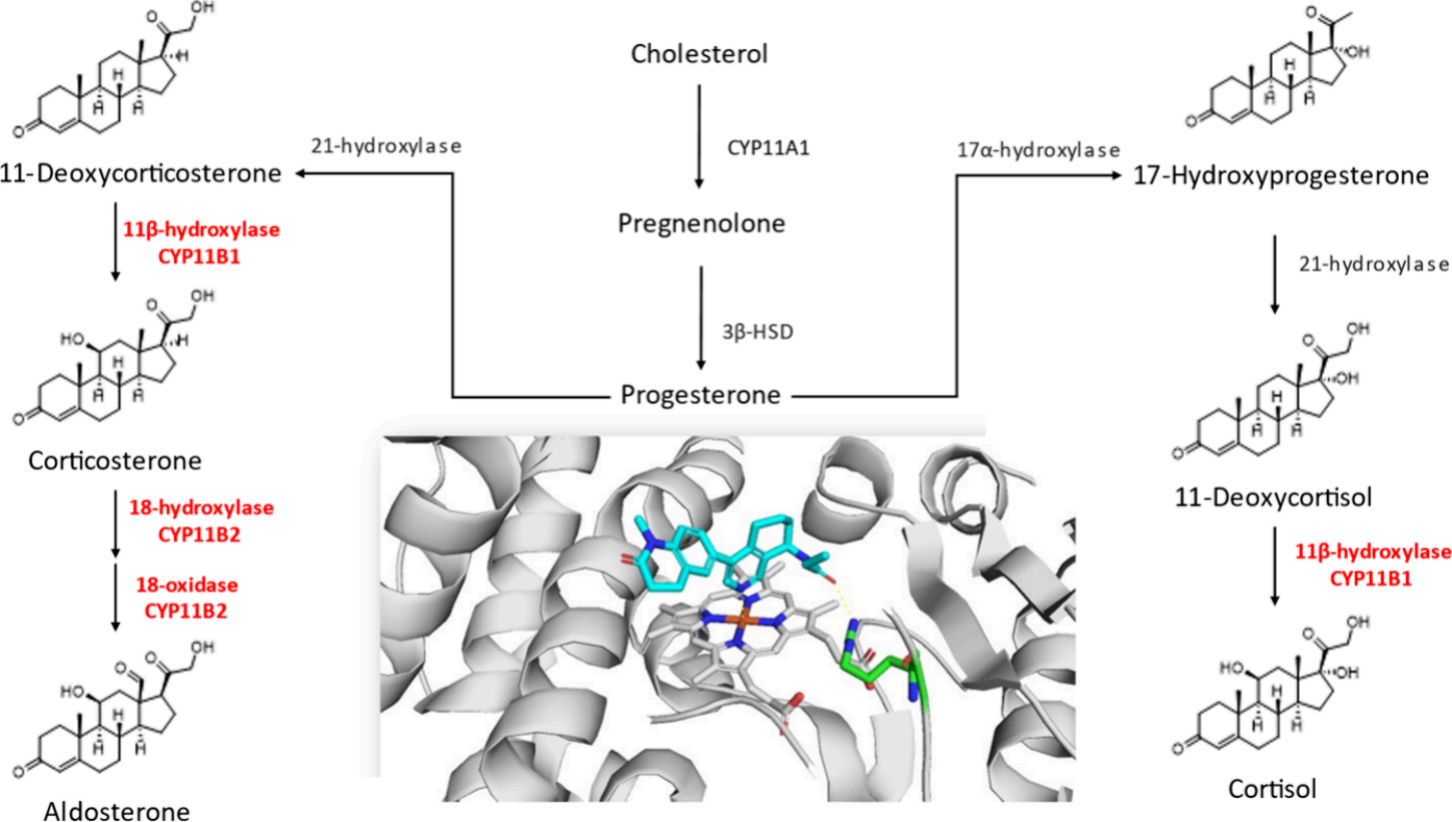
Biosynthetic pathway of aldosterone and cortisol and modeling of Baxdrostat into CYP11B2. Selective inhibition of CYP11B2 reduces aldosterone synthesis (left), while preserving cortisol production (right). Baxdrostat binds CYP11B2 via N–Fe coordination of its pyridine ring within the heme pocket and hydrogen bonding of the amide side chain to Arg110, supporting its high specificity. The Baxdrostat–CYP11B2 complex shown in Figure 2 represents a structure-based molecular docking model derived from previously reported CYP11B2 structural templates (pdb: 4fdh) and is intended to illustrate a plausible binding mode rather than an experimentally determined complex. The model was generated by the authors.

## Baxdrostat and the Discovery of Aldosterone Synthase Inhibitors

Attempts to pharmacologically suppress aldosterone synthesis have been explored for more than two decades. The first generation of inhibitors, including FAD-286 and its derivative LCI699 (later renamed Osilodrostat), demonstrated that it was possible to reduce circulating aldosterone levels by targeting CYP11B2.^2^ However, these compounds suffered from inadequate selectivity. Their inhibition of CYP11B1 suppressed cortisol production, resulting in adrenal insufficiency and limiting clinical development in hypertension. LCI699 ultimately found a therapeutic niche in Cushing’s disease, where cortisol suppression is beneficial, but it failed as an antihypertensive agent. Advances in structural biology helped illuminate the path forward. Crystallographic studies revealed that CYP11B2 possesses subtle active-site differences compared to CYP11B1, including unique orientations of residues such as Arg120 and hydrophobic cavity geometries that can be exploited to enhance selectivity. These insights enabled medicinal chemists to develop novel scaffolds with improved potency and specificity. Among the most promising were sulfonylpyrimidines, which demonstrated nanomolar inhibition of CYP11B2 with markedly reduced cross-reactivity to CYP11B1.^20,21^ Although not all chemotypes were translated successfully in vivo, this era of optimization laid out the groundwork for Baxdrostat. Baxdrostat (CIN-107,RO6836191) emerged from high-throughput screening campaigns and iterative medicinal chemistry. Its tetrahydroisoquinoline scaffold was optimized for both potency and selectivity, achieving more than 100-fold preference for CYP11B2 over CYP11B1.^22^ Importantly, Baxdrostat retained oral bioavailability and a favorable pharmacokinetic profile, including a plasma half-life of approximately 30 h, which allows convenient once-daily dosing. The compound demonstrated robust aldosterone suppression in preclinical models without cortisol inhibition, validating the long-sought hypothesis that selective aldosterone synthase inhibition is achievable (Figure 3). Selective inhibition of aldosterone synthase (CYP11B2) over the closely related steroidogenic isoform CYP11B1 represents a central medicinal chemistry challenge due to the very high sequence conservation between the two enzymes, particularly within the heme-proximal catalytic region. While the coordination of the heteroaromatic isoquinoline nitrogen of Baxdrostat to the heme iron is largely conserved between both isoforms, structure-guided homology modeling and crystallographic studies of early tetrahydroisoquinoline inhibitors have revealed that functional selectivity can be achieved through subtle differences in the extended hydrophobic binding cavity. Several nonconserved amino-acid residues located distal to the catalytic center modulate pocket geometry, plasticity, and substrate access channel topology, thereby influencing ligand orientation and productive binding modes. In particular, optimized aryl substitution patterns were found to progressively occupy a CYP11B2-preferred subpocket defined by residues such as Trp116 and Phe130, enabling favorable π-stacking interactions and steric complementarity that are less accessible in CYP11B1. These peripheral interactions appear to restrict conformational freedom required for cortisol-forming catalytic cycles, thereby reducing off-target inhibition of cortisol biosynthesis despite conserved heme anchoring. Collectively, these findings highlight that isoform selectivity within highly homologous cytochrome P450 enzymes can arise not only from direct active-site contacts but also from exploitation of distal structural divergences that shape ligand dynamics and catalytic competency. Beyond achieving structural isoform selectivity at the molecular level, the successful clinical translation of Baxdrostat also required careful optimization of physicochemical and pharmacokinetic properties to ensure that potent CYP11B2 inhibition could be maintained in vivo without compromising metabolic stability or functional selectivity. Optimization of the tetrahydroisoquinoline scaffold further illustrates how potency and metabolic stability can be partially decoupled through rational medicinal chemistry design. Progressive rigidification of the ligand framework, combined with strategic aryl substitution, improved both microsomal stability and oral exposure while preserving high affinity for CYP11B2. Lipophilicity tuning played a particularly important role in balancing target engagement with favorable pharmacokinetics, as moderate increases in logD enhanced permeability and binding efficiency but required careful control to avoid solubility limitations and excessive metabolic clearance. In addition, pronounced enantioselective differences in inhibitory potency demonstrated that stereochemical control of ligand orientation within the enzyme pocket could significantly enhance selectivity without increasing molecular size or lipophilicity. These combined strategies enabled the identification of candidates with sustained plasma exposure and a pharmacodynamic window in which free drug concentrations remained sufficient to suppress aldosterone synthesis while largely avoiding inhibition of cortisol production. Such exposure-driven functional selectivity highlights the importance of integrating structure-based design with ADME optimization when targeting highly conserved cytochrome P450 isoforms.

**Figure 3:**
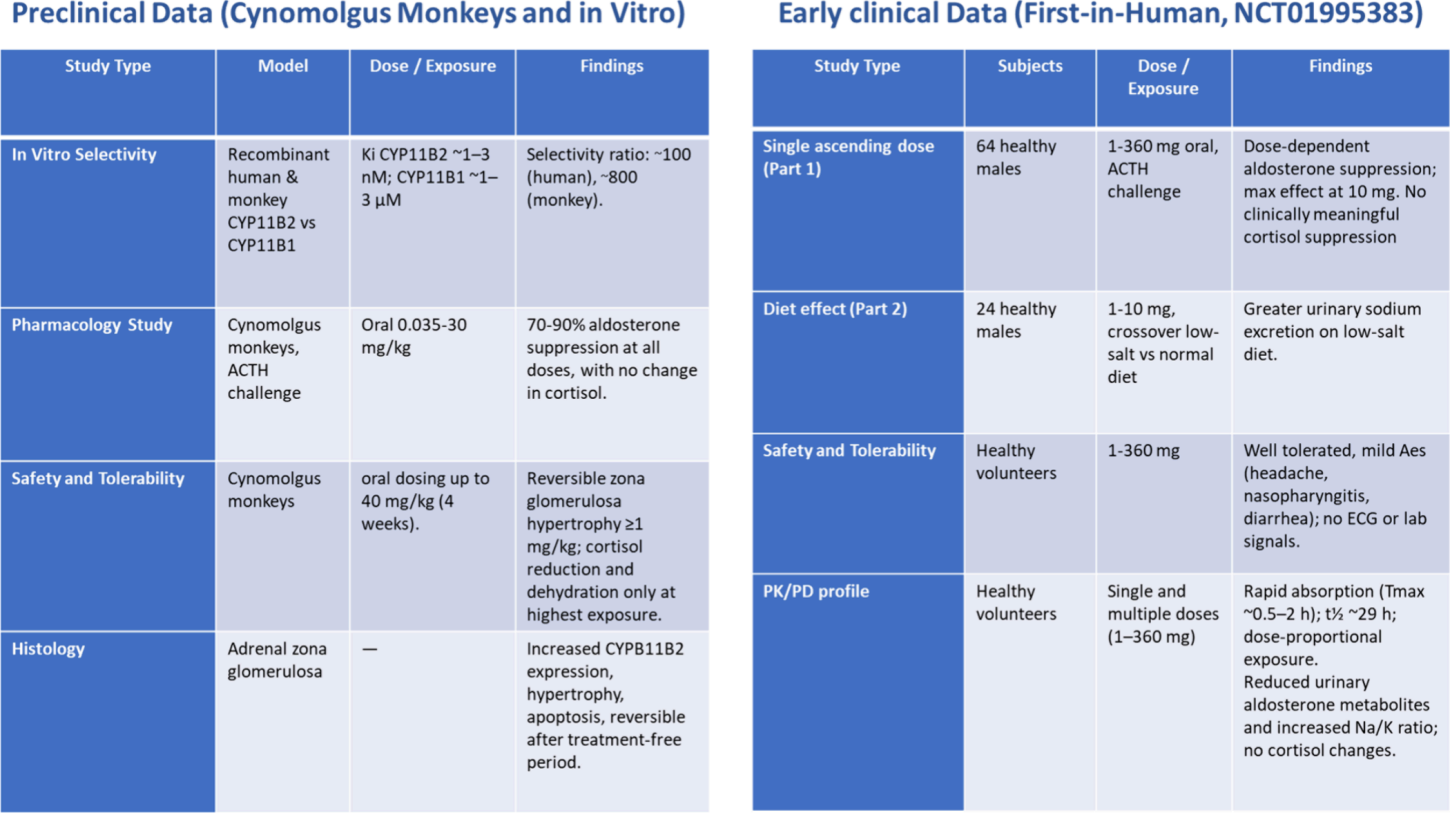
Overview of preclinical pharmacodynamic effects, first-in-human dose escalation outcomes, and safety observations supporting selective CYP11B2 inhibition by Baxdrostat.

## Preclinical and Early Clinical Development

In nonhuman primates, Baxdrostat produced a dose-dependent reduction in aldosterone synthesis in response to adrenocorticotropic hormone (ACTH) challenge while sparing cortisol production. These studies also noted histological changes in the adrenal zona glomerulosa, such as hypertrophy and increased expression of steroidogenic enzymes, findings consistent with the expected pharmacological blockade of CYP11B2. Importantly, these changes were partially reversible after cessation of therapy, suggesting an acceptable safety margin (Figure 3).^23^ First-in-human studies confirmed Baxdrostat’s pharmacological selectivity. In healthy volunteers, single and multiple ascending doses across a 360-fold range produced marked reductions in plasma and urinary aldosterone concentrations while leaving cortisol unaffected, even at doses far exceeding those required for efficacy. Pharmacodynamic markers such as 11-deoxycorticosterone and 11-deoxycortisol rose only at supratherapeutic exposures, further demonstrating the compound’s selectivity. Electrolyte balance was maintained, with hyperkalemia observed only rarely and at high exposures. Together, these data established proof-of-concept that Baxdrostat could safely suppress aldosterone biosynthesis in humans (Figure 3).^23^ Compared to spironolactone, Baxdrostat appeared to avoid many of the endocrine side effects—such as gynecomastia and menstrual irregularities—that limit the tolerability of mineralocorticoid receptor antagonists. Preliminary results also suggest a potentially lower burden of electrolyte disturbances, although the risk of hyperkalemia remains and requires careful monitoring.

## Clinical Development of Baxdrostat

Encouraged by preclinical and early human findings, Baxdrostat advanced into randomized clinical trials. The BrigHTN study, a phase 2 trial in patients with resistant hypertension, demonstrated significant reductions in seated systolic blood pressure compared with placebo. Conversely, the HALO trial in patients with uncontrolled but nonresistant hypertension failed to reach its primary end point, suggesting that Baxdrostat’s efficacy is most pronounced in the setting of pathophysiological aldosterone excess (Figure 4). These early studies underscored the importance of careful patient selection and trial design.^24^ The pivotal BaxHTN phase 3 trial, recently published in the New England Journal of Medicine, enrolled nearly 800 patients across multiple countries.^25^ Participants with uncontrolled or resistant hypertension were randomized to receive 1 mg or 2 mg of Baxdrostat or placebo in addition to background therapy. After 12 weeks of treatment, patients in both Baxdrostat arms achieved substantial reductions in systolic blood pressure, averaging 14.5 and 15.7 mmHg respectively, compared to 5.8 mmHg in the placebo group. The placebo-adjusted reductions of 8.7–9.8 mmHg were statistically significant and clinically meaningful. Safety data revealed hyperkalemia in a small percentage of patients (2–3%), which was manageable with monitoring and did not outweigh the overall benefit-risk profile.

**Figure 4:**
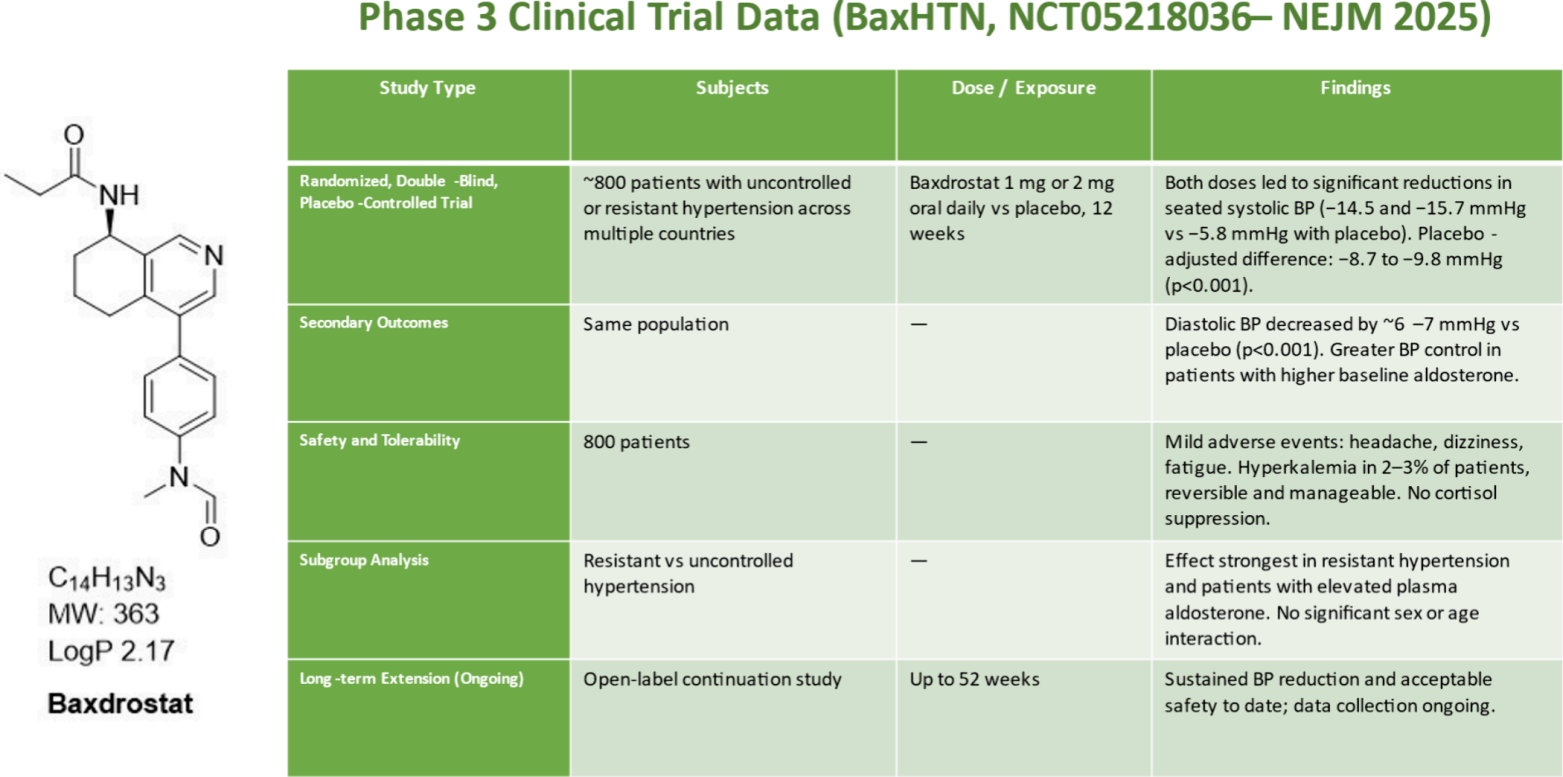
Chemical structure of Baxdrostat and summary of clinical blood pressure reductions observed in phase 3 trials.

These results confirm that Baxdrostat is the first-in-class aldosterone synthase inhibitor capable of delivering robust antihypertensive efficacy in a population with a critical unmet medical need.^25^

## Clinical Impact and Perspectives

The advent of Baxdrostat represents a milestone in the treatment of hypertension. For decades, the notion of selectively targeting CYP11B2 was met with skepticism, given its near-identical homology to CYP11B1. Baxdrostat demonstrates that careful structural exploitation of subtle enzymatic differences, combined with persistent medicinal chemistry optimization, can overcome such obstacles.^26^ The success of this program establishes CYP11B2 as a validated druggable target and heralds the emergence of an entirely new therapeutic class. Beyond resistant hypertension, Baxdrostat is likely to find application in conditions where aldosterone excess contributes to pathophysiology. Primary aldosteronism, long underdiagnosed, affects a substantial fraction of hypertensive patients and confers disproportionate cardiovascular risk. Direct aldosterone synthase inhibition may also prove valuable in chronic kidney disease, where aldosterone drives fibrosis and accelerates disease progression. Heart failure with preserved ejection fraction (HFpEF), a syndrome characterized by diastolic dysfunction and often linked to aldosterone-mediated fibrosis, represents another potential arena for Baxdrostat.

Nevertheless, important questions remain. The long-term safety of chronic aldosterone suppression must be clarified, including potential impacts on adrenal morphology, stress responses, and electrolyte homeostasis. Hyperkalemia, while manageable in trials, will need careful monitoring in clinical practice. Equally critical will be the development of strategies to identify patients most likely to benefit, possibly through biomarker-based selection or genetic screening. From a broader perspective, Baxdrostat’s success may inspire renewed exploration of other steroidogenic cytochrome P450 enzymes as drug targets (e.g., CYP4Z1 for cancer). These enzymes play central roles in endocrine physiology and pathophysiology, and their selective inhibition could unlock novel therapeutic strategies across cardiovascular, metabolic, and oncologic diseases.^17^ Ultimately, Baxdrostat should not be viewed merely as another antihypertensive agent, but as an example of how upstream, enzyme-targeted modulation of aldosterone biology may redefine therapeutic strategies.

## Conclusions

Baxdrostat represents a structurally rational and mechanistically precise innovation in hypertension therapy. If ongoing and future studies confirm durable efficacy and long-term safety, it has the potential to inaugurate a new era of precision pharmacology in cardiovascular medicine, much as ACE inhibitors and angiotensin receptor blockers reshaped practice in the past. Its emergence highlights a broader shift toward direct aldosterone synthase inhibition, with several analogues and next-generation compounds already under investigation - though structural data remain limited. This growing competitive landscape not only validates the therapeutic concept but also raises expectations for demonstrating long-term safety, differentiation from existing agents, and tangible clinical benefits.^17^ Whether Baxdrostat retains its lead role or is succeeded by improved analogues, the field is clearly moving toward a new paradigm in cardiovascular pharmacology - one defined by mechanism-based, highly selective interventions.^27^ Ultimately, the story of Baxdrostat is not just that of a single molecule, but of a broader transition in how we address the endocrine underpinnings of cardiovascular disease.
